# Comparison of Membrane Targeting Strategies for the Accumulation of the Human Immunodeficiency Virus p24 Protein in Transgenic Tobacco

**DOI:** 10.3390/ijms140713241

**Published:** 2013-06-26

**Authors:** Goretti Virgili-López, Markus Langhans, Julia Bubeck, Emanuela Pedrazzini, Guillaume Gouzerh, Jean-Marc Neuhaus, David G. Robinson, Alessandro Vitale

**Affiliations:** 1 Department of Plant Cell Biology, Centre for Organismal Studies, University of Heidelberg, Heidelberg D-69120, Germany; E-Mails: goretti_virgili@yahoo.es (G.V.-L.); markus.langhans@cos.uni-heidelberg.de (M.L.); julia.bubeck@urz.uni-heidelberg.de (J.B.); 2 Institute of Agricultural Biology and Biotechnology, National Research Council (CNR), via Bassini 15, Milano 20133, Italy; E-Mail: pedrazzini@ibba.cnr.it; 3 Laboratory of Cell and Molecular Biology, University of Neuchatel, Rue Emile-Argand 11, Neuchâtel CH-2000, Switzerland; E-Mail: guillaume.gouzerh@unine.ch

**Keywords:** HIV p24, transgenic plants, membrane proteins, endomembrane system, protein targeting, thrombin cleavage

## Abstract

Membrane anchorage was tested as a strategy to accumulate recombinant proteins in transgenic plants. Transmembrane domains of different lengths and topology were fused to the cytosolic HIV antigen p24, to promote endoplasmic reticulum (ER) residence or traffic to distal compartments of the secretory pathway in transgenic tobacco. Fusions to a domain of the maize seed storage protein γ-zein were also expressed, as a reference strategy that leads to very high stability via the formation of large polymers in the ER lumen. Although all the membrane anchored constructs were less stable compared to the zein fusions, residence at the ER membrane either as a type I fusion (where the p24 sequence is luminal) or a tail-anchored fusion (where the p24 sequence is cytosolic) resulted in much higher stability than delivery to the plasma membrane or intermediate traffic compartments. Delivery to the tonoplast was never observed. The inclusion of a thrombin cleavage site allowed for the quantitative *in vitro* recovery of p24 from all constructs. These results point to the ER as suitable compartment for the accumulation of membrane-anchored recombinant proteins in plants.

## 1. Introduction

The demand for large-scale production capacities of recombinant pharmaceuticals requires new strategies of production. Bacteria, yeast and mammalian cell cultures have been the preferred bioreactors, but alternative, more economically-viable strategies are desirable for those proteins for which the potential demand is highest, *i.e.*, bulk pharmaceuticals. Plants present several advantages in this regard: they produce biomass at relatively low costs that can be further reduced by easy scaling-up, they grow faster than transgenic animals and present much lower risks of contamination from human pathogens [1,2].

Protein stability, which is one of the bottlenecks for the high production of recombinant proteins in plants [2–4], can be manipulated by changing the subcellular localization of a given protein. Insertion into the secretory pathway followed by retention in the endoplasmic reticulum (ER), using either the K/HDEL receptor mechanism or, more recently, the formation of insoluble protein bodies, represent successful strategies to increase by one or two orders of magnitude the accumulation of cytosolic or secreted proteins [5,6]. It has also been shown by pulse-chase analysis that the higher accumulation achieved in these ways is indeed due to increased half-life [7,8]. The attachment of immunoglobulin domains [9] or elastin-like sequences [10–12] have also resulted in improved accumulation, possibly because of the formation of polymers or gel-like structures within the lumen of the ER-membrane system [13].

The use of transmembrane domains (TMD) to anchor proteins to membranes is an alternative approach that is receiving growing attention. The examples that we will summarize below involve oleosin, which is the major membrane protein of plant oil bodies, membrane-bound IgG (mIgG, which function as surface antigen receptors thanks to a TMD present in the heavy chain), the TMD of the plant vacuolar sorting receptor BP80/VSR, and the tail-anchor of type IV membrane proteins. In oilseeds, accumulation of triacylglycerols occurs between the phospholipid bilayer of the ER membrane, giving rise to oil bodies, which are therefore compartments with an unusual single-layer limiting membrane. Oleosin has a peculiar conformation, with the *N*- and *C*- termini exposed into the cytosol and a membrane-embedded central region [14]. Thus, hirudin, a thrombin-inhibitor peptide, and insulin have been expressed in seeds of oilseed rape and Arabidopsis, respectively, as cytosolic fusions to the *C*-terminus of oleosin [15,16]. Accumulation of the hirudin fusion was about 1% of the total soluble protein (TSP) and that of insulin 0.1% of TSP. In young tobacco leaves, mIgG (coexpressed heavy and light chain) accumulated on the plasma membrane and represented about 1.1% of total protein, a value similar to that of secreted IgG [17]. However when the constant regions of the heavy chain, which include the TMD, were fused to antigen binding regions of a microcystin-specific antibody, accumulation reached only 0.005% of total protein, a much lower level compared to the 0.6% reached upon expression of the original, secreted microcystin-specific antibody [18]. Members of the BP80/VSR family are type I transmembrane proteins [19] and are responsible for the sorting of many soluble vacuolar proteins to the plant vacuole [20,21]. A proof-of-concept study showed that the TMD and cytosolic tail of the pea BP80 receptor, placed at the *C*-terminus of a secretory form of YFP, allows the delivery of YFP to protein storage vacuoles in tobacco seeds [22]. Substitution of the BP80 cytosolic tail with that of αTIP, an aquaporin characteristic of the tonoplast of storage vacuoles, did not change the result. Accumulation in seeds was between 0.5% and 5% TSP. Vacuolar sorting probably occurred also in vegetative tissues, but accumulation was not measured [22]. Finally, many proteins that perform their function on the cytosolic side of membranes are post-translationally attached to the bilayer thanks to a *C*-terminal TMD “tail” and are thus termed tail-anchored (TA), or type IV integral membrane proteins [23]. Thus, the *C*-terminal TA of human cytochrome b5 has been successfully used to accumulate proteins at the ER of plant cells without impairing cellular function and has markedly increased the accumulation of the cytosolic HIV antigen Nef when compared to expression of the natural, soluble form [24,25].

A further refinement would be to use sequences mediating interaction with a particular membrane through its intrinsic properties. One such property is membrane thickness, which has been shown to differ significantly in different compartments of the secretory system of animal cells. TMD length of ER, Golgi and plasma membrane proteins has thus been found to correlate significantly with membrane thickness [26]. The same property has also been demonstrated for plant cells using GFP as a passenger protein [27]. It is thus possible to choose the target membrane by modifying the TM length.

The human immunodeficiency virus (HIV) structural protein p24 is a major candidate subunit to be included in a vaccine against HIV, because of the strong association between p24 and immune response to HIV infection, decreased viral load and better disease outcome [28,29]. p24 has been expressed as a soluble protein in a number of plant subcellular localizations [9,30–33]. With the aim of assessing the suitability of different membranes of the plant endomembrane system for the accumulation of foreign protein, in the present study p24 was expressed in transgenic tobacco as a type I integral membrane protein with TMD domains of different length, and as a TA-protein. The *in vivo* stability and accumulation of these constructs were determined and compared with those of p24 fusions with the *N*-terminal portion of γ-zein, a maize ER protein-body forming storage protein [34,35].

## 2. Results

### 2.1. Accumulation of p24 Engineered into either Type I or TA Integral Membrane Protein, or Fused to the *N*-Terminal Domain of γ-Zein

**Figure 1:**
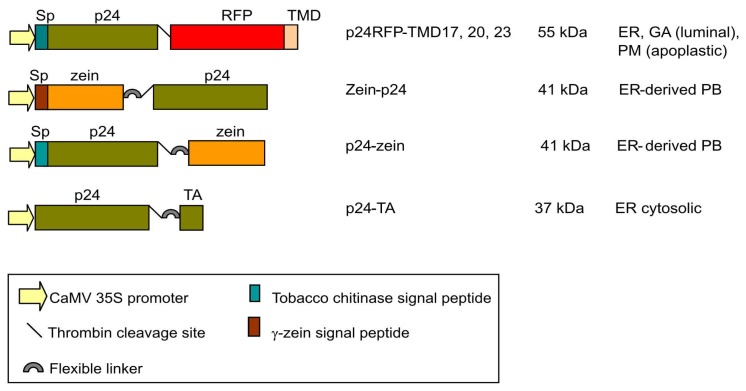
Schematic presentation of the expression cassettes used in this study. Full length HIV-1 p24 was used in all the constructs. The expected molecular masses of the fusion proteins are given as well as the expected localization(s) and the topology of the p24 sequence (luminal or apoplastic) in the fusions with TMDs. The zein fusions are expected to be luminal. ER: endoplasmic reticulum, GA: Golgi apparatus, PM: plasma membrane, PB: protein bodies, TA: tail-anchor.

The p24 sequence was fused to targeting domains that have previously been tested in other chimeric proteins (Figure 1). To anchor p24 to membranes of the endomembrane system as a type I integral membrane protein, the following fusion construct was generated, starting from the *N*-terminus: the signal peptide from tobacco chitinase for insertion into the ER, the p24 sequence, the thrombin cleavage site LVPRG, the red fluorescent protein (RFP) to allow detection by fluorescence microscopy, and the TMD23 sequence used by Brandizzi *et al.* [27]. This construct, named p24-RFP-TMD23, was expected to reach the plasma membrane, because a secretory form of GFP (*i.e.*, GFP preceded by a signal peptide) fused to TMD23 is also delivered to the plasma membrane [27]. Two additional constructs were generated in which the hydrophobic sequence of TMD23 was shortened from 23 to 20 or 17 amino acids, thus producing p24-RFP-TMD20 and p24-RFP-TMD17. When assayed with the above-mentioned GFP tag, these variants localized in the Golgi apparatus and in the ER, respectively [27]. Thus, in all of the above-described p24 constructs, the p24 and RFP sequences were expected to be inserted co-translationally into the ER lumen, be anchored there by the TMD and then be retained in the ER (TMD17) or proceed along the secretory pathway depending on TMD length. The strategy to anchor p24 at the ER membrane maintaining its natural exposure to the cytosolic environment was very similar to that used to produce Nef-TA [25]: the p24 sequence was followed by the thrombin cleavage site, the flexible linker (GGGGS)_3_, the TMD of the TA protein rabbit cytochrome b_5_ with its polar flanking regions and, finally the OP3 epitope containing an *N*-glycosylation consensus site (Figure 1, p24-TA). The *C*-terminal polar sequence and the OP3 epitope are the only regions expected to be translocated into the ER lumen. Finally, in an effort to promote protein-body formation to be used as a reference for high stability in the ER, the fusion construct zein-p24 contains the first 112 amino acids of γ-zein, including its 19aa signal peptide, the flexible linker, the thrombin cleavage site and, in *C*-terminal position, the p24 sequence (Figure 1). The zein portion includes six cysteine residues as well as a repeated, amphipathic sequence. These features promote the formation of large polymers that are insoluble unless treated with reducing agents [34–36]. To test for a possible position effect of the zein sequence, a second construct was produced (p24-zein), with the following structure: tobacco chitinase signal peptide, p24 sequence, thrombin cleavage site, flexible linker, 89 amino acids of γ-zein starting from the fifth residue after its signal peptide cleavage site. The latter is exactly the same γ-zein fragment used to produce zeolin [34].

**Figure 2:**
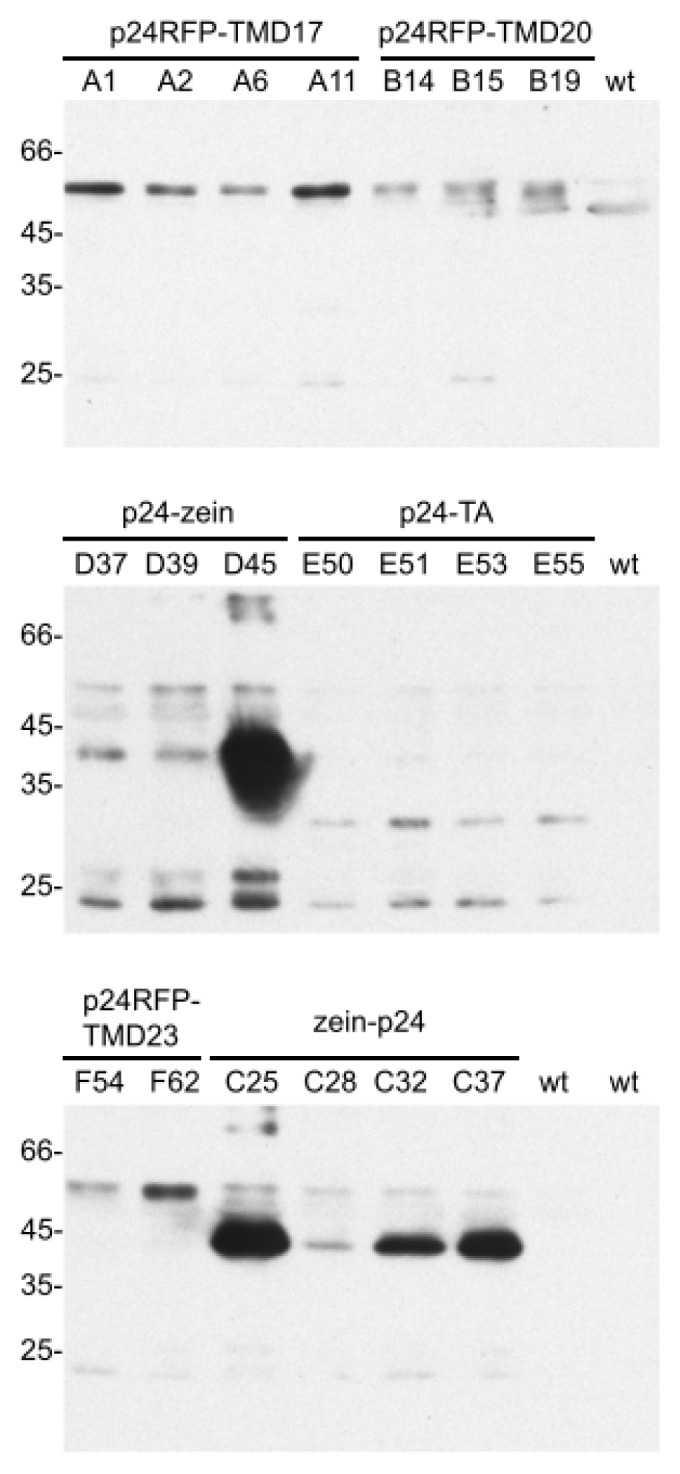
Protein accumulation. Proteins were extracted from young leaves (4–7 cm-long) excised from transgenic tobacco plants expressing the indicated constructs or from wild type (wt) plants. Aliquots corresponding to 10 μg total protein were analyzed by SDS-PAGE and protein blot with p24 antibodies. For each construct, each individual lane contains the extract from a different independent transgenic plant. Numbers at left indicate the positions of molecular mass markers, in kDa.

The six hybrid construct cassettes were placed under the control of the CaMV 35S promoter and used to produce transgenic *Nicotiana tabacum* plants. For each construct, 13 to 17 independent hygromycin-resistant plantlets were obtained from 25 to 30 independent callus clones. Six week-old leaves from selected T0 plants or from wild type plants, as control, were homogenized in the presence of non-ionic detergent and reducing agent. Total protein extracts were analyzed by western blotting with sheep p24 antibodies (Figure 2). Occasionally the antiserum recognized a polypeptide migrating just above the 45 kDa marker, which could be detected also in control extracts (Figure 2, top panel, wt) and possibly corresponds to the very abundant Rubisco large subunit. Apart from this, the antiserum specifically recognized recombinant polypeptides of the expected apparent molecular mass in the transgenic plants, but not in controls: around 55, 30, and 40 kDa for the type I integral membrane proteins, the TA anchored and the zein fusions, respectively (Figure 2). Accumulation varied in individual plants, especially in the case of the zein fusions, which in most cases were very abundant when compared to the other p24 fusions. An additional polypeptide around 25 kDa was also often detected, strongly suggesting proteolysis leading to the release of the p24 moiety. Comparison of its relative amount with that of the respective intact fusion indicated that the event generating the 25 kDa polypeptide was particularly evident in p24-TA plants (Figure 2, middle panel).

In plants that accumulated high amounts of the zein fusions, products larger than the 66 kDa marker were also detected (see lines D45 and C25), probably representing dimers of the 40 kDa polypeptide. Consistently, when other chimeric proteins containing the *N*-terminal γ-zein domain were expressed in transgenic plants, reducing agents caused solubilization but incomplete disassembly: dimers and larger polymers remained detectable by SDS-PAGE [8,34,35], possibly due to strong hydrophobic homotypic interactions caused by the amphipathic repeats [37].

**Figure 3:**
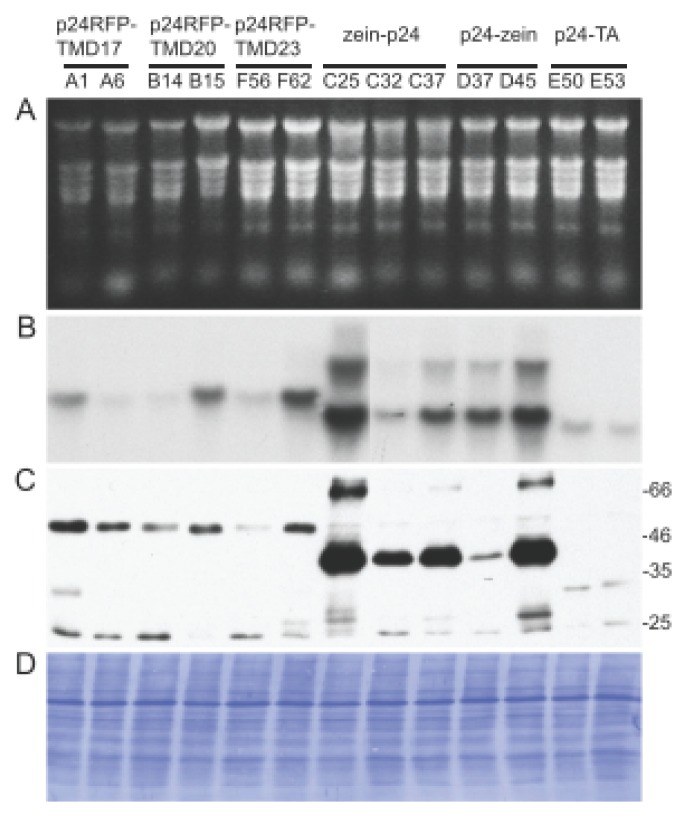
Protein and mRNA accumulation in leaves of transgenic tobacco plants expressing different p24 constructs. Young leaves (4–7 cm-long) were excised from transgenic tobacco plants expressing the indicated p24 constructs. Proteins and mRNA were extracted and equal amounts of material was analyzed for each of the different transgenic lines. Analysis was by RNA agarose electrophoresis followed by staining (**A**) and RNA blotting using a p24 probe (**B**); SDS-PAGE followed by protein blot with p24 antiserum; (**C**) and coomassie blue staining to detect total protein (**D**). Numbers at right of panel C indicate the positions of molecular mass markers, in kDa.

To verify whether variability in protein accumulation could be related to mRNA levels, leaves were excised and each individual leaf divided into two parts, to extract either proteins or RNA. Analysis was then performed using northern blotting with a p24-specific probe or western blotting with the p24 antibodies (Figure 3). In general, the highest mRNA levels were observed for the zein fusions and the lowest for the TA fusions (Figure 3B). Besides the major transcript of the expected size (around 1.1 kb) an additional, larger component was also detected in plants transformed with the zein fusions, probably because of secondary structures formed as a consequence of the high GC content of the zein gene [38–40]. When plants with high or low expression for each construct were compared, there was a correspondence between higher mRNA and higher protein accumulation (Figure 3, compare panels B and C). When similar levels of recombinant mRNA could be considered, the zein fusions were those accumulating higher amount of protein, followed by the TMD17 fusion, whereas the other fusions resulted in lower accumulation.

**Figure 4:**
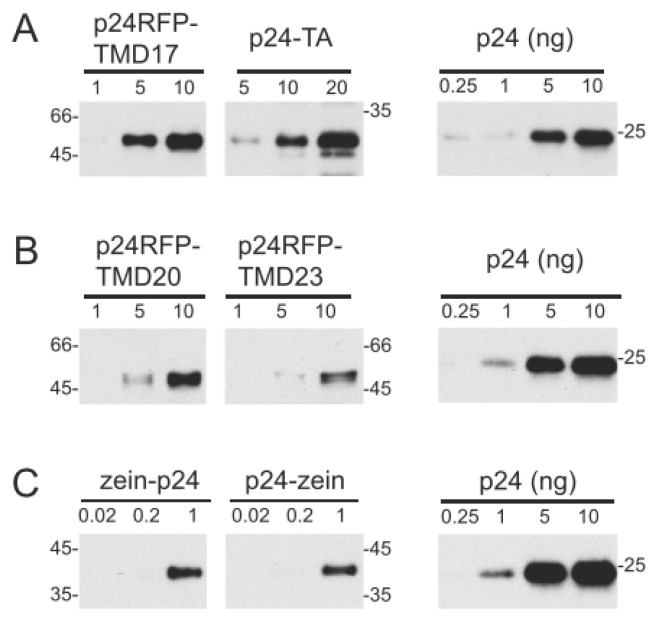
Quantification of the p 24 fusions accumulated in transgenic tobacco. Total proteins were extracted from leaves of plants expressing the indicated constructs. The amount of total extract indicated in each lane (in μg) was analyzed by SDS-PAGE and western blot with p24 antibodies. Each panel (**A**, **B** and **C**) is derived from a western blot in which also the indicated amounts (in ng) of recombinant, pure p24 were loaded as a reference. Numbers at left or right indicate the positions of molecular mass markers, in kDa.

**Figure 5:**
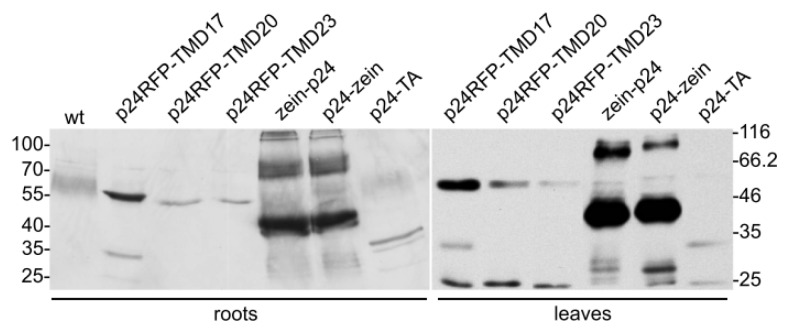
Comparison of p24 fusions accumulated in roots and leaves of transgenic tobacco. Total proteins were extracted from roots or leaves of plants expressing the indicated constructs or from wild type (wt) plant. Extract containing 20 or 10 μg of total soluble protein, from roots or leaves respectively, were separated by SDS-PAGE and immunodetected using sheep anti-p24 antibodies. Numbers indicate the position of molecular mass markers, in kDa.

The best-accumulating plant for each construct was selected for further analysis. Semi-quantitative comparison by protein blotting, using three dilutions each of the leaf extracts and a purified p24 as standard of known concentration (Figure 4), indicated that the intact recombinant protein represented around 0.3% TSP in the p24-RFP-TMD17 plant, compared to 0.15% TSP in the p24-RFP-TMD20 and p24-RFP-TMD23 plant. Accumulation of p24-TA was very similar to those of p24-RFP-TMD20 and -TMD23 (about 0.15% TSP), in spite of its relatively lower transcript level. In the best accumulating plants, both zein-p24 and p24-zein represented about 1% TSP. It should be noted that this could be an underestimation, because the higher molecular mass polymers were not considered in the quantification. For each plant, relative levels of accumulation of the recombinant protein were very similar in leaves and roots (Figure 5). The SDS-PAGE banding patterns were also similar, but the additional polypeptide around 25 kDa was detected in much higher amounts in leaves, strongly suggesting higher proteolysis leading to the release of the p24 moiety.

Altogether, the ratios between mRNA and protein levels suggest highest stability of the zein fusions and lowest stability of the proteins destined to the Golgi complex and plasma membrane. Anchorage to the ER membrane as type I or TA proteins seemed to promote intermediate stabilities. To verify these hypotheses, pulse-chase analysis was performed.

### 2.2. *In Vivo* Protein Stability

**Figure 6:**
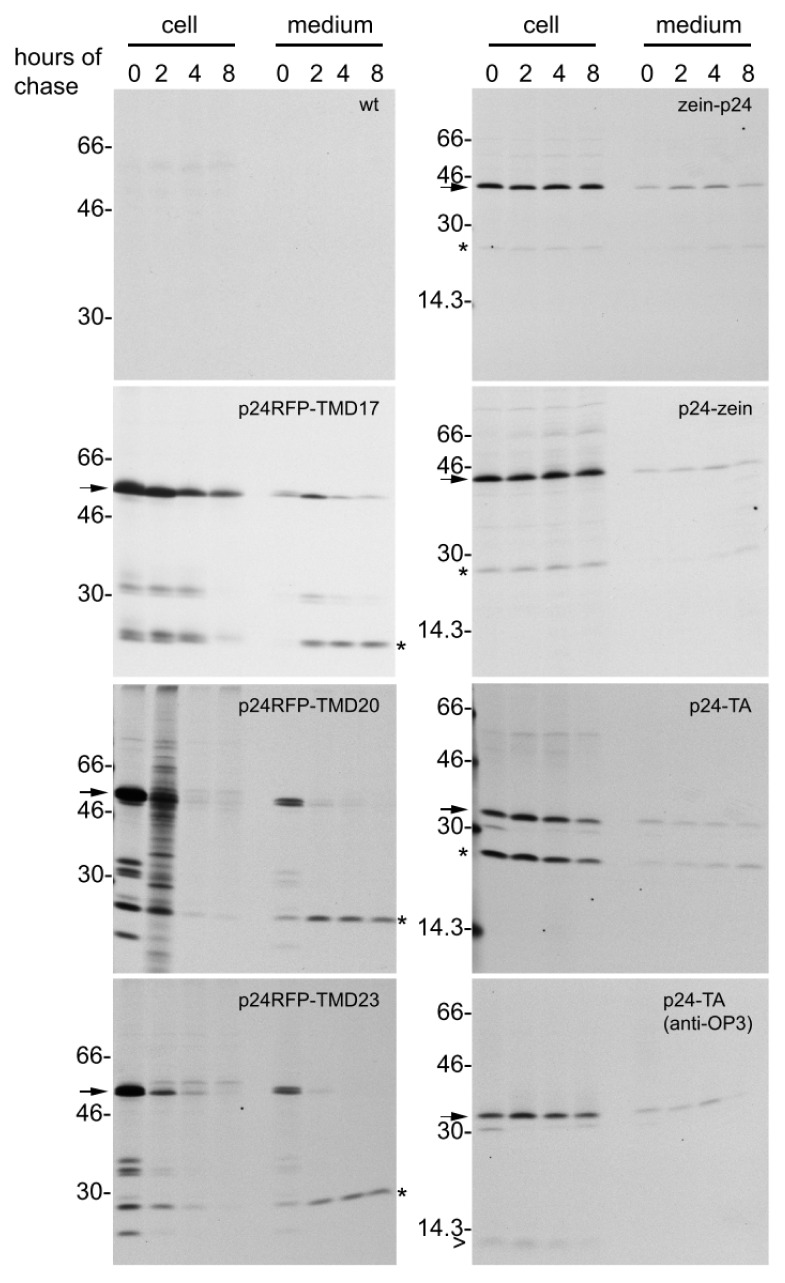
*In vivo* protein stability. Tobacco leaf protoplasts were prepared from transgenic plants expressing the indicated fusion proteins or from wild type (wt) plants. Protoplasts were pulse-labeled for 1 h with [^35^S] Met and [^35^S] Cys and subjected to chase for the indicated times. Protoplast (cell) or incubation media (medium) homogenates were subjected to immunoprecipitation with rabbit p24 antibodies, and analyzed by SDS-PAGE followed by fluorography. The positions of each intact fusion protein (arrow), of the 25 kDa fragment (asterisk) and the fragment recognized by the OP3 antibodies (arrowhead) are indicated. In each panel, numbers at left indicate the position of molecular mass markers, in kDa.

Protoplasts were prepared from young leaves of the same plants used to measure protein accumulation and were subjected to 1 h pulse-labeling with a mixture of ^35^S methionine and cysteine, followed by different chase time-points in the presence of an excess of non-radioactive amino acids. After homogenization, cells or incubation media were subjected to immunoselection with rabbit anti-p24 antiserum (the sheep polyclonal antibodies used for western blot were unable to immunoselect the undenatured p24 fusions from cell homogenates). The immunoselected proteins were analyzed by SDS-PAGE followed by radiofluorography (Figure 6). At 0 h chase, a major radioactive component with molecular mass around 55 kDa was immunoselected from p24-RFP-TMD17 protoplast homogenate (arrow) but not from wild type (wt) tobacco protoplasts, indicating that this component represents the intact chimeric protein. The recovery of intact p24-RFP-TMD17 decreased during the chase, but after 8 h chase the intact polypeptide was still clearly detectable. Conversely, newly synthesized p24-RFP-TMD20 and p24-RFP-TMD23 (arrows) became almost completely undetectable already after 4 h chase, indicating that they are much less stable than the TMD17 fusion.

As expected for membrane-anchored proteins, no intact transmembrane type I p24 chimeric form was secreted (Figure 6, panels at left, medium). The faint signals around 55 kDa, detectable in the medium for each construct, did not increase consistently during the chase, indicating lack of secretion and minor contamination from protoplasts during medium recovery, which is very difficult to avoid in these experiments. On the other hand, lower molecular weight fragments with apparent molecular mass around 35 and 25 kDa were detected in the cell samples. The 25 kDa fragment increased in amount in the medium between 0 and 2 h chase, strongly suggesting that it was indeed secreted and originated from post-translational proteolysis, either within the endomembrane system or at the apoplast after the fusion protein reached in part the plasma membrane. The 25 kDa polypeptide (asterisks) most probably corresponds to the one detected also by protein blot. Because of the topology of the p24-TMD constructs, p24 could be released by apoplastic proteases; alternatively, p24 released intracellularly by proteolytic events would be expected to remain luminal and therefore available for default secretion through the secretory pathway. From these pulse-chase experiments, it can therefore be concluded that the higher accumulation of p24-RFP-TM17 in comparison to the two other p24 constructs is due to higher protein stability in the ER, but that all constructs release minor amounts of a soluble p24-containg fragment.

The stability of intact p24-TA (Figure 6, p24-TA panel, arrow) was similar to that of p24-RFP-TMD17, and therefore markedly higher when compared to p24-RFP-TMD20 or p24-RFP-TMD23. Altogether protein blot and pulse-chase analysis indicate that the addition of a TA leads to an accumulation comparable to that observed for p24-RFP-TMD20 and p24-RFP-TMD23, which are much less stable proteins. This is probably due to the particularly low accumulation of the TA transcripts. The 25 kDa polypeptide was also immunoprecipitated, in relatively high amounts (Figure 6, p24-TA panel, asterisk). This is in agreement with its relatively high accumulation revealed by protein blotting. This polypeptide was present in high amounts also at the end of the pulse; its relative proportion with respect to intact p24-TA did not change during the chase. This 25 kDa polypeptide could originate either from premature termination of translation or a very early *in vivo* proteolytic event. Although we cannot not rule out low levels of premature translation termination, the second hypothesis is favored by the observation that *in vivo* immunodetection with OP3 antibodies revealed the presence of small amounts of a polypeptide of about 13 kDa, in addition to the intact p24-TA (Figure 6, anti-OP3 panel, arrowhead). This most probably corresponds to the *C*-terminal portion of the recombinant protein. Early proteolysis could occur on a proportion of p24-TA molecules that fail to anchor to the membrane and remain fully cytosolic: anchoring to the membrane was not fully efficient also in the case of Nef-TA fusion protein [25].

Zein-p24 and p24-zein did not undergo marked degradation during 8 h chase, being therefore much more stable than any of the other fusions (Figure 6, zein-p24 and p24-zein panels, arrow). The intact polypeptides present in the medium did not increase during the chase and most probably represent contamination from cells, whereas a very minor amount of what could be free p24 (around 25 kDa, asterisk) increased in the medium during the chase. Therefore, intact zein was not secreted, but a very minor proportion of molecules underwent a proteolytic event followed by secretion. It can thus be concluded that the enhanced accumulation of the zein fusions with respect to the TMD fusions, can be largely ascribed to markedly improved protein stability.

### 2.3. Subcellular Localization

**Figure 7:**
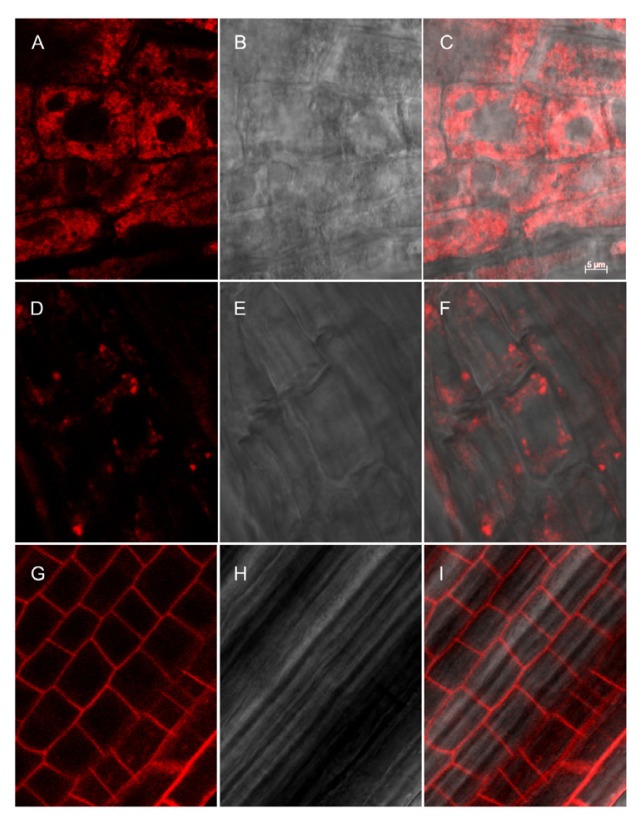
Subcellular localization of fluorescent recombinant proteins in roots of transgenic tobacco. Root epidermis from plants expressing p24RFP-TMD17 (**A**–**C**), p24RFP-TMD20 (**D**–**F**) or p24RFP-TMD23; (**G**–**I**) were analyzed by confocal microscopy. RFP fluorescence (**A**, **D** and **G**), brightfield (**B**, **E** and **H**) and merge (**C**, **F** and **I**) are shown. Scale bar: 5 μm.

The presence of RFP in the type I constructs allowed us to examine their localization by confocal microscopy. In root epidermis, p24-RFP-TMD17 was detected as a reticulate pattern, strongly suggesting ER localization (Figure 7A–C), whereas p24-RFP-TMD23 showed a typical plasma membrane labeling (Figure 7G–I). p24-RFP-TMD20 was present as small intensely labeled dots over a much less intense reticular background (Figure 7D–F). The dots could represent Golgi cisternae, the trans-Golgi network (TGN) or late endosomes, which in plants are represented by the prevacuolar compartment/multivesicular bodies (MVB). The patterns were apparently in agreement with the results obtained by Brandizzi *et al.* [27] using GFP fusions with the same TMDs.

**Figure 8:**
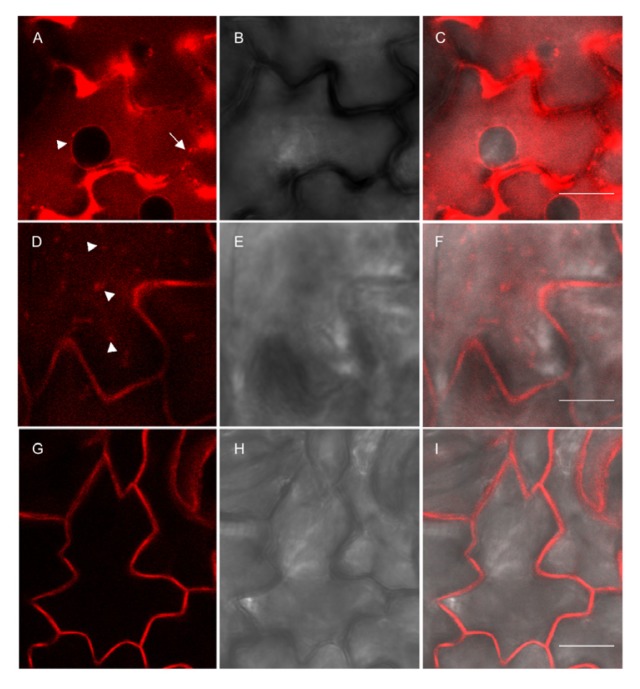
Subcellular localization of fluorescent recombinant proteins in leaves of transgenic tobacco. Leaf epidermis from plants expressing p24RFP-TMD17 (**A**–**C**), p24RFP-TMD20 (**D-F**) or p24RFP-TMD23; (**G**–**I**) were analyzed by confocal microscopy. RFP fluorescence (**A**, **D** and **G**), brightfield (**B**, **E** and **H**) and merge (**C**, **F** and **I**) are shown. Arrows indicate the nuclear envelope and putative cortical ER; arrowheads indicate intracellular dots. Scale bars: 10 μm.

In leaf epidermal cells, signals from all constructs were present at the cell surface (Figure 8). This suggests at least partial traffic to the plasma membrane of all the type I fusions or early proteolytic events leading to secretion of a soluble fragment containing RFP. As discussed above, the recovery of the 25 kDa fragment (which most probably represents the p24 moiety) in the protoplast incubation medium upon pulse-chase analysis also indicates proteolytic events. Red fluorescence seems in some cases to be present in the apoplast, suggesting that not only the p24 moiety but also free RFP is released by proteolysis. In addition to the cell surface, the TMD17 and TMD20 proteins also labeled internal structures: p24-RFP-TMD17 was clearly detected at the nuclear envelope (Figure 8A, arrowhead), which is continuous with the ER network, and in what could be cortical ER (Figure 8A, arrow); p24-RFP-TMD20 labeled cytoplasmic dots (Figure 8D, arrowheads). No other labeling besides the cell surface was instead detectable upon p24-RFP-TMD23 expression (Figure 8, G–I). The results in Figure 8 thus suggest that also in leaves the different type I constructs are present in part at the expected subcellular location, and indicate that correct sorting is however not fully efficient, consistently with the existence of proteolytic events already revealed by pulse-chase.

**Figure 9:**
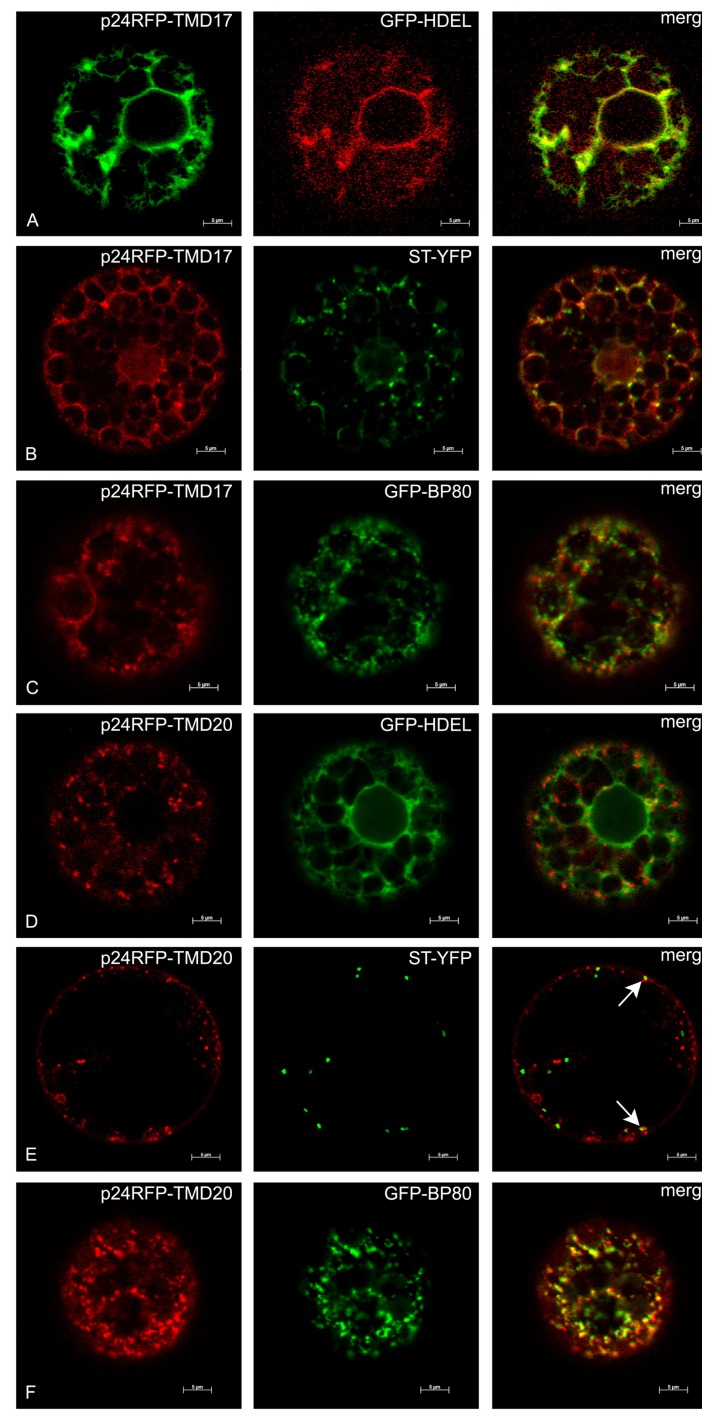
Subcellular localization of p24RFP-TMD17 and p24RFP-TMD20 in transiently transformed protoplasts. Each horizontal set of images (**A**–**F**) shows the indicated p24 construct transiently co-expressed in tobacco protoplasts for 24 h with markers for the ER (GFP-HDEL), trans-Golgi cisternae (ST-YFP) or TGN/MVB (GFP-BP80). Arrows in panel **E**, merge, indicate colocalization. Scale bars: 5 μm.

**Figure 10:**
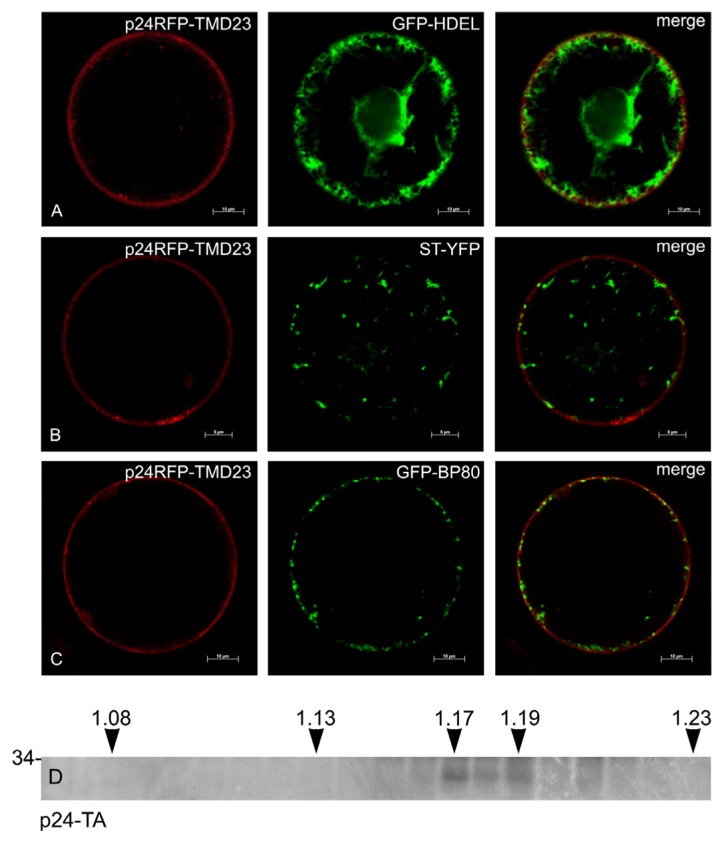
Subcellular localization of p24RFP-TMD23 and p24-TA. **A**, **B** and **C**: p24RFP-TMD23 was transiently co-expressed in tobacco protoplasts for 24 h with markers for the ER (GFP-HDEL), trans-Golgi cisternae (ST-YFP) or TGN/MVB (GFP-BP80). Scale bars: 10 μm (**A** and **C**); 5 μm (**B**). (**D**) Leaves of transgenic tobacco expressing p24-TA were homogenized in the absence of detergent. Subcellular compartments were separated by ultracentrifugation on isopycnic sucrose gradient. Proteins in each fraction were finally analyzed by SDS-PAGE and protein blot with antibodies against p24. Top of the gradient is at left; numbers on top indicate fraction density (g/mL). The number at left indicates the position of molecular mass marker, in kDa.

To analyze more precisely the subcellular localization of the different type I constructs, these were transiently co-expressed in tobacco protoplasts together with the following markers of subcellular compartments: GFP-HDEL for the ER [41], sialyl transferase-YFP (ST-YFP) for the trans-Golgi cisternae [27], GFP-BP80 for the TGN and MVB [42]. p24-RFP-TMD17 mainly co-localized with GFP-HDEL (Figure 9A). A few dots highlighted by p24-RFP-TMD17 were also superimposed to the Golgi (Figure 9B) and TGN/MVB (Figure 9C) markers, consistent with minor escape from the ER along the secretory pathway. p24RFP-TMD20 did not accumulate in the ER (Figure 9D), occasionally colocalized with the Golgi marker (Figure 9E, arrows) and much more frequently with the TGN/MVB marker (Figure 9F). p24RFP-TMD23 clearly and specifically labeled the cell surface and did not colocalize with any of the markers we used (Figure 10A–C). In conclusion, transient expression confirmed the ER and plasma membrane localization of p24RFP-TMD17 and p24RFP-TMD23, respectively and indicated that most probably the majority of p24RFP-TMD20 localized at the TGN or MVB, with little retention at the Golgi-cisternae.

To verify the subcellular localization of p24-TA, young leaves were homogenized in the absence of detergent and subjected to isopycnic centrifugation on continuous sucrose gradient. This was performed in the presence of magnesium, to preserve the interactions between polysomes and the ER membrane and thus allow a better separation of ER-derived microsomes from other compartments. Each fraction was then analyzed by SDS-PAGE and protein blot (Figure 10D). The fractions containing p24-TA peaked between 1.17 and 1.19, the expected density of the rough ER [43].

### 2.4. *In Vitro* Release of p24 upon Thrombin Cleavage

**Figure 11:**
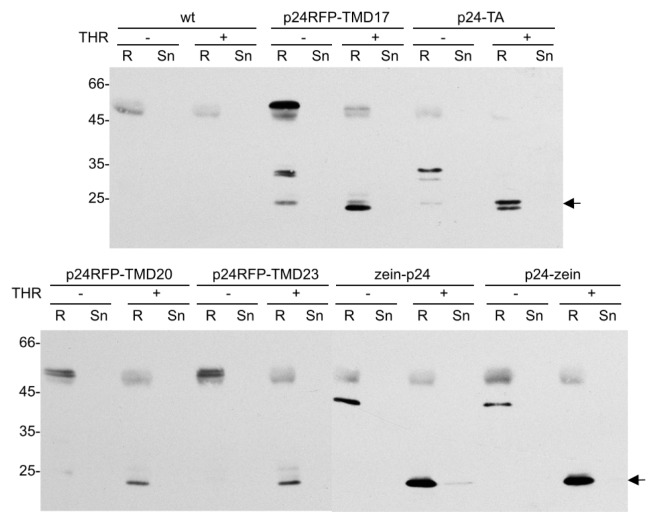
*In vitro* release of p24 from the fusion proteins by thrombin cleavage. Leaf homogenates from transgenic tobacco expressing the indicated p24 fusions or wild type (wt) tobacco plants were immunoselected with rabbit p24 antibodies and protein A-Sepharose. The beads were then split into two equal aliquots, which were incubated with thrombin (THR+) or with buffer without enzyme as control (THR−). After centrifugation, the resin pellet (R) and supernatant (Sn) were then subjected to SDS–PAGE and protein blot using sheep anti-p24 antibodies. In each panel, the arrow at right indicates the position of p24 released by thrombin. Numbers at left indicate the position of molecular mass marker, in kDa.

Since the aim of this study was to verify new strategies for the production of proteins of pharmaceutical interest, we finally tested whether p24 could be released *in vitro* from the fusion constructs by thrombin cleavage at the engineered site. Frozen leaf material was therefore homogenized in extraction buffer containing 0.2% Triton X-100. For the zein fusions, the reducing agent 2-mercaptoethanol was also included, because disulfide bonds make the zein fusions insoluble [34]. The p24 fusion proteins were then immunoprecipitated with protein A-Sepharose beads coated with rabbit p24 antibodies. The beads were then washed, resuspended and split into two portions that were incubated overnight in the presence or absence of thrombin. The samples were then centrifuged and the supernatants and pellets denatured and analyzed by SDS-PAGE followed by immunoblotting (Figure 11). Release was quantitative, and for each construct the fusion protein was recovered in the untreated bead sample (THR−, R) while free p24 (arrow) was recovered in the treated bead sample (THR+, R).

## 3. Discussion

We have shown here that in leaves of transgenic tobacco a cytosolic protein can accumulate to levels corresponding to or higher than 0.1% when expressed as fusions that anchor it to the luminal or cytosolic side of endomembranes. We have also shown that engineering of a thrombin cleavage site in all the recombinant fusions can be used to recover the protein of interest by *in vitro* proteolytic treatment.

In previous studies, accumulation of the natural, cytosolic soluble form of p24 in leaves of transgenic tobacco or Arabidopsis reached about 0.35% of TSP or 0.2 mg/g fresh weight [30,31]. In comparison, introduction of p24 into the secretory pathway in tobacco by the addition of a signal peptide led to accumulation to about 0.1% TSP [9]. In the latter case, pulse-chase analysis of leaf protoplasts indicated that the protein was efficiently secreted, with no signs of intra- or extracellular degradation during 5 h chase. Accumulation was however enhanced about 13-fold through fusion of this secretory form of p24 to the constant α2 and α3 regions of the α heavy chain of immunoglobulin A. The increased accumulation was most probably due to the formation of dimers, promoted by the Ig domains, and an inhibition of secretion, suggesting that the secreted construct undergoes degradation in the apoplast [9]. These experiments demonstrated that the lumen of the ER is not an adverse environment for the folding and therefore stability of newly synthesized p24, unlike what was observed for Nef, another cytosolic HIV antigen [8,44].

### 3.1. TMD Length and Subcellular Localization

It has been previously demonstrated that the length of the TMD is a determinant for the subcellular localization of type I integral membrane GFP fusions in plant cells [27]. We have shown here that this targeting mechanism can be exploited to deliver a cytosolic viral antigen to the ER, the plasma membrane or intermediate compartments of the plant cell endomembrane system. The localizations of the of the TMD17 and TMD23 p24 fusions were those expected from the results previously obtained using GFP as a TMD reporter [27]. It is thus conceivable that this technology can be applied to other recombinant proteins as well. Our results also indicate that there is higher escape of the TMD17 and TMD20 fusions to the cell surface in leaf cells than in root cells. Genes encoding proteins of the secretory pathway machineries are usually expressed at much higher levels in roots than leaves [45,46], an expected feature given the known high secretory activity of growing roots. We can therefore hypothesize that overloading of the mechanism that mediates TMD length-based localization is more likely to occur by recombinant protein expression in leaves than roots.

It was previously shown that GFP-TMD20 is mainly located in the Golgi cisternae [27]. Transient co-expression with compartment markers indicated that p24-RFP-TMD20 is instead only marginally retained in the Golgi complex and mainly resides at the TGN and/or MVB. It thus seems that, when fused to p24, TMD20 allows a further traffic step beyond the Golgi cisternae compared to the simpler GFP fusion. We do not know whether this is due to an influence of the appended protein on the way the TMD is positioned within the lipid bilayer or to a different behavior in protoplasts as opposed to leaves. However, we wish to underline that both GFP-TMD20 [27] and p24-RFP-TMD20 are clearly competent for ER exit and incompetent for efficient delivery to the plasma membrane.

Delivery to the tonoplast was not observed for any construct, consistently with the current view that tonoplast sorting requires specific motifs located in cytosolic domains [47]. In the absence of such motifs, type I membrane proteins with a TMD of sufficient length reach the plasma membrane.

Pulse-chase revealed that *in vivo* stability of p24-RFP-TMD17 is at least twice those of p24-RFP-TMD20 and p24-RFP-TMD23. This led to higher accumulation, thus extending to membrane proteins the previous conclusions that established the ER as the best compartment of the endomembrane system for the accumulation of non-membrane recombinant proteins in vegetative plant tissues [5]. The lower stability of p24-RFP-TMD20 and p24-RFP-TMD23 compared to the ER-located fusion indicates susceptibility to proteases located either at the apoplast or along the endomembrane system. The fact that p24-RFP-TMD20 is as unstable as the plasma membrane construct was not expected by us. As a tentative explanation, the known existence of Golgi-located proteases can be taken into account [48], as well as the possibility that retention in intermediate compartments of secretory protein traffic may be less stringent than ER retention and therefore a higher proportion of p24-RFP-TMD20 polypeptides may reach the cell surface.

### 3.2. Attachment to the Cytosolic Face of the ER

Attachment of p24 to the cytosolic side of the ER membrane through fusion to a TA resulted in accumulation to about 0.1% TSP, which is lower than the corresponding ER-localized type I fusion p24-RFP-TMD17. It should however be noticed that pulse-chase analysis indicated similar half lives of the two constructs and suggested that a relevant proportion of p24-TA could fail correct anchoring to the membrane. Such a failure would not allow for *N*-glycosylation of the *C*-terminal tail of p24-TA, which follows the TMD. Indeed, a newly synthesized polypeptide slightly smaller than p24-TA is clearly detectable at 0h chase inside cells and rapidly disappears during the chase (Figure 6, p24-TA, minor polypeptide comigrating with the 30 kDa marker). This unstable polypeptide contains the OP-3 epitope, suggesting that it is full-length, unglycosylated p24-TA that failed insertion into the membrane (Figure 6, p24-TA, anti-OP-3). Very similarly, newly-synthesized Nef-TA molecules that were demonstrated to fail anchoring were not glycosylated, had a slightly lower mobility than the membrane-anchored polypeptides and were unstable [25]. Therefore, the lower accumulation of p24-TA is most likely due both to its lower transcript levels and to inefficient anchoring.

Obviously, the environments on the two sides of the ER membrane are very different. All TA proteins are exposed to the cytosol. We have shown that TA anchorage to the ER membrane markedly increases the accumulation of Nef, another cytosolic HIV antigen, thus indicating that membrane attachment influences cytosolic degradation [25]. However, it is not known whether and how the specific membrane in which a given TA protein resides influences its half life [49]. It will thus be interesting to establish whether fusion of p24 to TA of increased lengths would affect stability.

### 3.3. Fusion to the Zein Domain

Fusion to domains of the maize prolamin γ-zein has emerged as a very promising strategy for the high accumulation of recombinant proteins [8,34,35]. The repeated and the proline-rich regions of γ-zein promote the formation of insoluble, ER-located protein bodies formed because of inter-chain disulfide bonds and hydrophobic interactions [34,50]. This strategy seems unable to rescue proteins that have severe folding defects or are unable for less clear reasons to undergo the zein-promoted insolubilization process [8,51] but, as discussed above, soluble p24 is not severely misfolded when introduced into the secretory pathway. Fusion to the zein sequences resulted in accumulations of to up to 1% TSP, which is several fold higher than the best integral membrane fusion we have produced. Pulse-chase studies showed very high stability of both zein-p24 and p24-zein, indicating that the relative position of the prolamin sequence is not relevant, suggesting independent folding of the two components of the fusion molecule. SDS-PAGE revealed the formation of polymers, a characteristic of protein bodies [8,34]. We have previously shown that zeolin, a chimeric zein-phaseolin fusion, accumulates to markedly higher levels than phaseolin-KDEL, suggesting that protein body formation provides higher stability compared to the retention mechanism used by soluble ER residents [34]. The results reported here also indicate a similar improvement with respect to ER residence for type I integral membrane and TA proteins, and support our hypothesis that protein bodies, besides being highly resistant to proteolytic attack [36] are largely excluded from the physiological mechanisms of turnover of soluble or membrane ER proteins [8,34].

### 3.4. Comparison with Other Strategies

Immunoglobulins are very stable in the plant apoplast and can accumulate to very high amounts [52], but they are more an exception than the rule in regard to stability of unmodified foreign proteins in transgenic plants. The other available examples of very high recombinant protein accumulation in leaves of transgenic plants (*i.e.*, above 5% TSP) are the result of the formation of very large polymers in the ER by protein engineering, or of high transcript levels, obtained either by transplastomic technology or the use of particularly strong, tissue specific promoters. Transplastomic technology has recently also been applied to p24, to a chimeric p24-Nef fusion and to the entire HIV Gag gene, which synthesizes the polyprotein precursor that includes p24 [32,33,53]. Accumulation levels well above 5% TSP, and reaching as much as 40% in the case of p24-Nef [33], were always related to extremely high mRNA levels. This protein accumulation was highly influenced by leaf age [32,33]. Pulse-chase revealed that Gag was stable in the stromal environment for at least 8 h [53], but no information on the stability of the other constructs in the stroma is available. It should also be underlined that the stromal environment can adversely affect the folding of foreign proteins, thus leading to low accumulation, as it has been observed for certain secretory proteins expressed in transplastomic plants [54].

The p24 levels obtained with the techniques used in the present study can in theory be markedly increased by coupling them with strategies to enhance accumulation of nuclear-encoded mRNAs. Thus, we feel that procedures giving high transcript levels coupled to methods that provide high protein stability within the endomembrane system could be competitive with transplastomic technology in terms of protein accumulation. This also applies for a number of foreign proteins that are not of the secretory class, besides providing, for naturally secretory proteins, the correct environment for folding and post-translational modifications such as glycosylation. Finally, membrane anchoring can improve purification protocols. It has been shown that centrifugation allows simple, efficient recovery of oleosin-containing protein fusions, because oil bodies float in aqueous buffers [15]. The opposite behavior of microsomes, which precipitate upon centrifugation, can also be used to eliminate cytosolic and apoplastic proteins. The pelleted membranes can then be subjected to *in vitro* digestion with the protease specific for the engineered proteolytic site, leading to recovery of the protein of interest in a soluble form.

## 4. Experimental Section

### 4.1. Plasmid Construction and Protein Engineering

A derived version of BH10 strain HIV-1 p24 (Accession number M15654.1) was used in this work. Constructs containing the p24 sequence (UniProtKB/Swiss database number P0C6F2, region 133–363) were prepared according to standard molecular techniques. Other sequences used were those coding for the following protein domains or peptides: the signal peptide of tobacco chitinase; monomeric red fluorescent protein (mRFP); the thrombin cleavage site LVPRG; the flexible linker (GGGGS)_3_; the following membrane anchoring regions and short cytosolic sequences, as described in Brandizzi *et al.* [27]: STIEGREAEALIPIAVGGALAGLVLIVLIAYLVGRKRS (TMD23), STIEGREAEALIPIAVGGALAGLVLIAYLVGRKRS (TMD20), STIEGREAEALIPIAVGGAL AGLAYLVGRKRS (TMD17). To construct p24-TA, a BamH1 restriction site and a thrombin cleavage site/linker followed by a SalI restriction site were appended to the 5′- and 3′- of the p24 coding sequence, respectively. The resulting p24linker was cloned into the BamHI/SalI sites of pDHA::cytb5 [25], which contains the *C*-terminal end of cyt b5 and the *N*-terminal sequence of bovine opsin (OP3). The final clone in pDHA vector was called p24-TA. For stable transformation, the EcoRI expression cassette was excised from p24-TA and subcloned into the dephosphorylated EcoRI site of pGreen0179. The construct was called p24TAG0179. To construct zein p-24, the first 112 amino acids of 37 kDa γ-zein, including its 19aa signal peptide were used; for p24-zein, 89 amino acids of γ-zein starting from the fifth residue after its signal peptide cleavage site were used (this is the same fragment used to construct zeolin in Mainieri *et al.* [34]). The constructs were first cloned into the vector pGY1 for transient expression in protoplasts, then into pDHA and finally, for Agrobacterium-mediated transformations, they were transferred into the binary vector pGreen0179.

### 4.2. Production and Growth of Transgenic Plants

Transgenic *Nicotiana tabacum* (cv. Petit Havana SR1) plants were produced using the *A. tumefaciens* mediated transformation method. 1–2 cm discs from fully expanded tobacco leaves were co-incubated with *A. tumefaciens* carrying the plasmid of interest, for 5 min. Leaf discs were transferred on shoot-inducing medium (SIM) agar (MS salts containing 3% sucrose, 0.1 mg/L 6-benzylaminopurine; Duchefa Biochemie, (Haarlem, The Netherlands), underside down at 25 °C in 16 h of light. After 2 days, the leaf discs were transferred to MS agar containing 0.5 mg/L 6-benzylaminopurine, 0.1 mg/L α-naphthalene acetic acid, 50 mg/L hygromycin for the selection of transgenic shoots and 100 mg/L carbenicillin to prevent further growth of Agrobacterium. Elongated shoots were excised from calli and transferred on to 3% sucrose MS agar, 0.1 mg/L indole-3-acetic acid, 50 mg/L hygromycin and 100 mg/L carbenicillin. Selected transgenic plants were grown in axenic conditions without antibiotics in MS basal salt medium containing 0.8% (*w*/*v*) plant agar in sterile Magenta boxes at 24 °C constant temperature, 16 h light/8 h dark, and were propagated every 4 to 6 weeks.

### 4.3. Protein Extraction, Western Blot Analysis and Subcellular Fractionation

Leaves from 4–6-week old plants were homogenized in an ice-cold mortar with ice-cold homogenation buffer (0.1 M Tris-HCl pH 7.8, 0.2 M NaCl, 1 mM EDTA, 2% 2-mercaptoethanol and 0.2% Triton X-100) supplemented with Complete Protease Inhibitor Cocktail (Roche). The ratio of buffer/weight was 5:1. The homogenates were centrifuged at 5000× *g* for 10 min at 4 °C and the protein concentration was determined by Bradford assay using Bradford reagent (Sigma-Aldrich, St. Louis, MO, USA). Equal amounts of total protein from each sample were denatured with loading buffer containing SDS and 2-mercatoethanol and separated by on 15% SDS-PAGE. After electrophoresis proteins were transferred to polyvinylidene fluoride membrane (Polyscreen, PerkinElmer, Waltham, MA, USA) and blots were probed with polyclonal sheep p24 antibodies (Aalto BioReagents, Dublin, Ireland). Proteins were detected using a horse radish peroxidase-conjugated anti-sheep secondary antibody (Sigma-Aldrich) followed by chemiluminescence with Super-Signal West Pico Chemiluminescent Substrate (Pierce, Rockford, IL, USA). Subcellular fractionation on isopycnic sucrose gradient was performed as described [34] using a linear 20%–50% (*w*/*w*) sucrose gradient.

### 4.4. Total RNA Isolation and Northern Blot

Total RNA was isolated from leaf material of different transgenic lines using Trizol reagent (Invitrogen Life Technologies, Carlsbad, CA, USA), according to the manufacturer’s instructions and following the protocol described in Barbante *et al.* [25]. RNA concentration was determined spectrophotometrically and its quality was visually inspected by agarose-formaldehyde gel electrophoresis. For northern blot, which was performed essentially as described [55], the RNA samples were denatured in 50% formamide, 2.2 M formaldehyde, 20 mM MOPS (3-(*N*-morpholino)propanesulfonic acid) pH 7.0, 5 mM Na acetate and 1 mM EDTA and resolved in a 1% agarose gel containing 2.2 M formaldehyde for 3 h at 50 V. After electrophoresis, the RNA in the gel was transferred by capillarity into a nylon Hybond-N+ membrane (Amersham Biosciences, Little Chalfont, UK). The DNA probe was labeled with the DecaLabel DNA labeling kit (Fermentas, Vilnius, Lithuania) using α-32P-dCTP (Perkin-Elmer) following the manufacturer instructions.

### 4.5. Pulse-Chase and Immunoprecipitation

Pulse-chase radioactive labeling of protoplasts derived from transgenic plants was performed using Pro-Mix (a mixture of [^35^S]-Met and [^35^S]-Cys; Amersham Biosciences) as described [43]. Chase was performed by adding unlabelled methionine and cysteine to a final concentration of 10 mM and 5 mM respectively. Protoplasts were homogenized by adding 2 volumes of ice-cold homogenization buffer to frozen samples (HB:150 mM Tris-HCl pH 7.5, 150 mM NaCl, 1.5 mM EDTA, and 1.5% Triton X-100) supplemented with Complete protease inhibitor cocktail (Roche, Basel, Switzerland). Protoplasts from plants expressing the zein fusions were homogenized in HB supplemented with 4% (*v*/*v*) 2-mercaptoethanol (Sigma). Immunoselection was performed as described previously [25] using rabbit polyclonal p24 antibodies (ARP432, Medical Research Council AIDS Directed Programme, Potters Bar, UK) or monoclonal OP3 antibodies [56] followed by incubation with Immunopure immobilized protein A (for the p24 antibody) or protein G (for the OP3 antibody, Pierce). Immunocomplexes were denatured with loading buffer containing SDS and 2-mercaptoethanol and loaded on 15% acrylamide gel using Rainbow ^14^*C*-methylated proteins (Sigma-Aldrich) as molecular mass markers. Radioactive polypeptides were revealed by fluorography and dried gels were exposed using the intensifying screen BioMax Transcreen LE (Kodak, Rochester, NY, USA).

### 4.6. Confocal Microscopy

Tissues were observed under a Zeiss Axiovert LSM510 Meta microscope using the Plan-Neofluar 25×/0.8 corr DIC and the *C*-Apochromat 63×/1.2 W corr water immersion objectives. Special settings were designed for observing single-, and double-expression with different XFP-tagged constructs. Fluorescence was detected by the Metadetector using the main beam splitters HFT 458/514 and HFT 488/543. Fluorophores were excited by line switching in the multi-tracking mode of the microscope. GFP was excited at 488 nm and emission at 496–518 nm and YFP by excitation at 514 nm and emission at 529–550 nm, both with the Argon laser. The RFP was excited with the HeNe1 (helium-neon) laser at 543 nm and emission at 593–636 nm. The double detection of GFP/RFP was performed at excitation 488/543 nm and emission at 496–518 nm and 593–636 nm whereas the double detection of YFP/RFP was performed at excitation 514/543 and emission at 529–550 nm and 593–636 nm. Pinholes were adjusted to 1 Airy Unit for each wavelength. Images were post-processed using the Zeiss LSM Image Browser (version 4.2.0.121, Carl Zeiss, Oberkochen, Germany, 2010).

### 4.7. Transient Expression in Protoplasts

Preparation of tobacco leaf protoplasts was done as previously described [57]. Protoplasts were resuspended in electroporation buffer and centrifuged at 80× *g* for 10 min, and the pellet and the underlying medium were removed again. This procedure was repeated twice by reducing the volume of electroporation buffer. In a last step, protoplasts were resuspended in electroporation buffer at a concentration of 5 × 106 protoplasts/mL. A total volume of 500 μL of the protoplast suspension was mixed with an appropriate amount of plasmid DNA or mixtures of plasmids previously dissolved in 100 μL of electroporation buffer. Plasmids encoding GFP-HDEL [41], ST-YFP [27] or GFP-BP80 [42] were coexpressed with the different p24 constructs. Electroporation was performed using stainless steel electrodes at a distance of 3.5 mm using a complete exponential discharge of a 1000 mF capacitor charged at 160 V. After 30 min of absolute rest, electroporated protoplasts were removed from the cuvettes and transferred to 5-cm Petri dishes with 2 mL of transient expression (TEX) buffer [57]. Protoplasts were then incubated for 24 h at 25 °C in a dark chamber. The next day, the samples were placed into a 15-mL tube and centrifuged at 85 g for 5 min without break. The pellet was removed and the suspension was ready for observation by confocal microscopy.

### 4.8. Thrombin Digestion

Nine hundred micrograms of total protein extracts from transgenic and control plants were subjected to immunoselection with rabbit p24 antibody as described before, except for an additional wash with PBS. The immunocomplexes were resuspended in 200 μL of PBS with 0.2% Triton X-100, supplemented with 4 μL of thrombin protease solution (1 U/μL) (Amersham Biosciences) or 4 μL of PBS (as control) and incubated at 22 °C for 20 h under gentle agitation. After digestion, the resin beads were precipitated by centrifugation and the supernatant was recovered. Resins (which contain the recombinant protein released by thrombin digestion) and supernatants were then analyzed by SDS-PAGE and protein blot, using sheep anti-p24 (1:500) and peroxidase conjugated donkey anti-sheep secondary antibody.

## 5. Conclusions

Using HIV p24 as a model protein, the aim of this work has been to compare *in vivo* stability and final accumulation level of a foreign recombinant protein expressed in transgenic tobacco, by exploiting different membrane-anchoring strategies that lead to various localizations within the endomembrane system. Although these membrane-anchoring approaches are less efficient when compared to γ-zein domain fusions that promote polymerization within the ER lumen [34], targeting to the ER membrane results in relatively high stability and accumulation when compared to anchoring to other endomembranes, and to previously reported results on the expression of p24 as a soluble protein [30]. High stability was observed both when p24 was exposed to the cytosolic or luminal side of the ER membrane. This extends to the ER membrane the previous observations that the ER lumen of plant cells is a very safe environment for the accumulation of many foreign proteins [5]. Anchorage to one or the other side of the ER membrane can be particularly useful (i) to avoid luminal post-translational modifications that occur downstream the ER along the secretory pathway; (ii) to allow folding of cytosolic proteins in their native environment and at the same time escape proteasomal degradation; (iii) as an alternative to polymerization strategies, when these can interfere with proper folding of the protein of interest [8]; (iv) to facilitate purification of the protein of interest.
